# Association of Body Mass and Brain Activation during Gastric Distention: Implications for Obesity

**DOI:** 10.1371/journal.pone.0006847

**Published:** 2009-08-31

**Authors:** Dardo Tomasi, Gene-Jack Wang, Ruiliang Wang, Walter Backus, Allan Geliebter, Frank Telang, Millar C. Jayne, Christopher Wong, Joanna S. Fowler, Nora D. Volkow

**Affiliations:** 1 National Institute on Alcoholism and Alcohol Abuse, National Institutes of Health, Bethesda, Maryland, United States of America; 2 Medical Department, Brookhaven National Laboratory, Upton, New York, United States of America; 3 Department of Psychiatry, Mt Sinai School of Medicine, New York, New York, United States of America; 4 Depertment of Anesthesiology, SUNY, Stony Brook, New York, United States of America; 5 St. Luke's/Roosevelt Hospital, Columbia University, New York, New York, United States of America; 6 National Institute on Drug Abuse, National Institutes of Health, Bethesda, Maryland, United States of America; Sapienza University of Rome, Italy

## Abstract

**Background:**

Gastric distention (GD), as it occurs during meal ingestion, signals a full stomach and it is one of the key mechanisms controlling food intake. Previous studies on GD showed lower activation of the amygdala for subjects with higher body mass index (BMI). Since obese subjects have dopaminergic deficits that correlate negatively with BMI and the amygdala is innervated by dopamine neurons, we hypothesized that BMI would correlate negatively with activation not just in the amygdala but also in other dopaminergic brain regions (midbrain and hypothalamus).

**Methodology/Principal Findings:**

We used functional magnetic resonance imaging (fMRI) to evaluate brain activation during GD in 24 healthy subjects with BMI range of 20–39 kg/m^2^. Using multiple regression and cross-correlation analyses based on a family-wise error corrected threshold *P* = 0.05, we show that during slow GD to maximum volumes of 500 ml and 700 ml subjects with increased BMI had increased activation in cerebellum and left posterior insula, and decreased activation of dopaminergic (amygdala, midbrain, hypothalamus, thalamus) and serotonergic (pons) brain regions and anterior insula, regions that were functionally interconnected with one another.

**Conclusions:**

The negative correlation between BMI and BOLD responses to gastric distention in dopaminergic (midbrain, hypothalamus, amygdala, thalamus) and serotonergic (pons) brain regions is consistent with disruption of dopaminergic and serotonergic signaling in obesity. In contrast the positive correlation between BMI and BOLD responses in posterior insula and cerebellum suggests an opposing mechanism that promotes food intake in obese subjects that may underlie their ability to consume at once large food volumes despite increasing gastric distention.

## Introduction

Why obese people continue to eat even when their stomach is full? The role of the stomach in satiety is not well understood. The human stomach can hold up to 1.5 liters of food [1] and the distention of the mechanoreceptors in the stomach wall might control food intake [2] by activating satiety brain regions [3]. Studies in animals have shown that gastric load suppresses food intake as a function of caloric value and volume of food intake (reviewed in [2]), and the human sensation of fullness after a meal is associated with the volume of GD [4], [5].

Neuroimaging studies that used a balloon placed in the human stomach have shown that distention of the gastric wall activates cortical and subcortical brain regions [3], [5]–[7]. During fMRI, gastric balloons with sudden [7] or gradual [3] expansions activate a visceral network that include the somatosentory cortex and the inferior dorsolateral prefrontal cortex as well as amygdala and insula. However, the effects of body mass on brain activation during GD are largely unknown. There is only one study that assessed BMI effects on brain activation during GD that reported lower amygdala activation for subjects with increased BMI [3], suggesting amygdala hypo-activation in obesity; though, the sample did not include obese patients. Since the amygdala is innervated by dopamine (DA) neurons [8] and obese subjects have dopaminergic deficits that correlate negatively with BMI [9] we hypothesized that the blunted amygdalar activation during gradual GD in subjects with higher BMI [3] would reflect lower activation in midbrain (the brain region were DA neurons are located) and in DA innervated brain regions involved in feeding behaviors (hypothalamus). Thus during gradual GD, BMI would correlate negatively with activation not just in the amygdala but also in midbrain and hypothalamus.

To test this hypothesis we used blood oxygenation level dependent (BOLD) fMRI and a gradual GD paradigm [3]. The larger sample size (N = 24) used in this study includes published data from 18 non-obese subjects using a GD volume of 500 ml, and unpublished data from five obese patients. The present study further expands on the prior study by including two different balloon volumes (500 ml and 700 ml) to assess the sensitivity of neuronal responses to volume differences during GD. Here we show that subjects with higher BMI had lower activation in a dopaminergic network (midbrain, hypothalamus, amygdala, and thalamus) and higher activation in cerebellum and posterior insula. The cross-correlation of time-varying fMRI signals among these BMI-sensitive regions further support a blunted network response, as opposed to isolated regional effects, in obesity.

## Methods

### Subjects

Twenty-four non-smoking and right-handed healthy subjects [20 men; age 32.2±7.0 years, education: 14.4±2.3 years; BMI range 20–39 kg/m^2^, mean = 26.8±5.8 kg/m^2^; 11 lean subjects (BMI<25 kg/m^2^), 8 overweight subjects (25 kg/m^2^<BMI<30 kg/m^2^), and 5 obese subjects (30 kg/m^2^<BMI)] participated in the study. All participants provided written informed consent approved by the local Institutional Review Board (Stony Brook University's Committee on Research Involving Human Subjects, CORIHS). Subjects were screened carefully with a detailed medical history, physical and neurological examination and urine toxicology for psychotropic drugs to ensure they were healthy at the time of the study. Subjects were included in the study if they were (a) 18–65 years old and (b) able to understand and give informed consent. Subjects were excluded if they used (c) anorexic medications for weight loss in the past 6 months; had (d) positive urine results for psychoactive drugs or pregnancy, history of (e) dependence on alcohol or other drugs of abuse (caffeine>5 cups/day or nicotine>1 pack/day), (f) neurological disorders of central origin or major psychiatric disorders, (g) esophageal reflux, (h) uncontrolled cardiovascular disease, (i) diabetes or other uncontrolled endocrine disease, (j) acute or chronic medical illness that may affect brain function or (k) head trauma with loss of consciousness>30 min, any (l) medical conditions that may alter cerebral function or (m) contraindications for MRI (metallic or electronic implants, metallic tattoos in the neck/head, claustrophobia). Subjects were asked to have their last meal at 7 PM the evening before the day of the study and were scanned between 16 and 18 h after their last meal.

### Balloon insertion in the stomach

**Figure 1:**
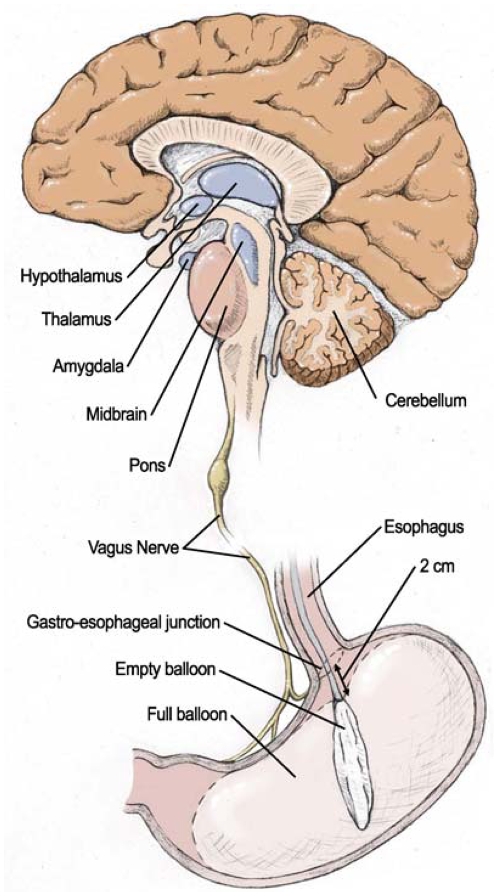
Gradual gastric distention (GD) paradigm. The deflated balloon was inserted orally and positioned in the stomach 2-cm above the gastro-esophageal junction. The solid and dashed lines depict the emptied (0 ml) and filled (500 ml for GD1 or 700 ml for GD2) balloon conditions. The balloon was filled and emptied with constant flow (5 ml/s) of tap water (warmed at 37°C) in either 90s to 500 ml (GD1) or in 140 s to 700 ml (GD2). The vagus nerve transmits the signal of a full stomach to the solitary and parabrachial nuclei in the brain stem that project to dopaminergic and serotonergic nuclei in midbrain and pons. Other regions implicated in the control of food intake are additionally highlighted (hypothalamus, amygdala, and cerebellum).

The balloon assembly consisted of a double-lumen tube (Fr-10) securely tied to a thin Latex non-lubricated condom with non-waxed dental floss [4]. The physician placed a small plastic mouthpiece coated with about 3 ml of 2% lidocaine viscous gel in the subject's mouth. A short time later (3 min or less), the mouthpiece was removed. Subjects were given a cup of lidocaine-water solution and they were asked to rinse the back of the tongue several times with it and spit it out. The deflated balloon assembly was orally introduced in the stomach by advancing gently the double-lumen tube. During this procedure, the subject was asked to swallow to facilitate placement of the balloon into the stomach. Then the balloon was filled with 100 ml of water at body temperature (∼37°C), using an electric pump (Masterflex L/S, Cole-Parmer Instrument Co., Vernon Hills, IL), and the tube was gently pulled out until resistance was met at the gastro esophageal junction. The tube was then pushed down another 2 cm to avoid obstructing esophageal flow (Fig. 1). The exterior end of the tube was taped to the cheek and shoulder to fix the balloon's position, and the water in the balloon was removed by using the electric pump with reversed water flow. The balloon insertion procedure was initiated between 15 and 17 h after the last meal.

### Gastric distention paradigm

After the balloon insertion, the subject was positioned in the MRI scanner. There were two different GD paradigms, GD1 and GD2, with different maximum distention volumes. Two filling-emptying cycles were used for both paradigms; after a 30 s-long resting baseline, the electric pump started a constant flow of water at body temperature (∼37°C) to fill up the balloon to either 500 ml in 90 seconds (GD1) or 700 ml in 140 s (GD2). The water flow was interrupted for 30 s when the volume reached maximum value (500 ml for GD1 and 700 ml for GD2). Then the balloon was emptied with reversed water flow. When the balloon reached null volume there was a pause for 30 s and the filling-emptying cycle was repeated to increase statistical power. The GD1 and GD2 fMRI runs were repeated once after a 1-minute resting interval to further increase statistical power. The total time of this fMRI paradigm was 45 minutes.

### Rating questionnaire

The subjects rated their fullness, discomfort, hunger, and desire for food during the last 16 s of the pauses [3]. These rating questions were displayed to the subjects using MRI-compatible goggles (MRVision2000, Resonance Technology Inc., Northridge, CA). The subjects responded by pressing one of the four available buttons in an MRI-compatible pad (Lumila LP-400, Cedrus Corporation, San Pedro, CA) [3]. The subjects were trained on how to use the response pad and how to respond to the questionnaire outside the MRI scanner and prior to the placement of the balloon in the stomach. The task was developed in E-prime (Psychology Software Tools, Inc., Pittsburgh, PA) and used a trigger pulse from the MRI console for precise synchronization with fMRI acquisition; subjects' responses were recorded in a standard PC using E-prime.

### Functional MRI: data acquisition

Subjects underwent MRI in a 4-Tesla whole-body Varian/Siemens MRI scanner. A T2*-weighted single-shot gradient-echo planar imaging (EPI) pulse sequence (TE/TR = 20/2000 ms, 4-mm slice thickness, 1-mm gap, 35 coronal slices, 64×64 matrix size, 3.1×3.1 mm in-plane resolution, 90°-flip angle, time points: 255 for GD1 or 355 for GD2, 200.00 kHz bandwidth) with ramp-sampling and whole brain coverage was used to collect functional images with BOLD contrast. Padding was used to minimize motion. Subject motion was monitored immediately after each fMRI run using a k-space motion detection algorithm [10] written in IDL (ITT Visual Information Solutions, Boulder, CO). Earplugs (−28 dB sound pressure level attenuation; Aearo Ear TaperFit 2; Aearo Company), headphones (−30 dB sound pressure level attenuation; Commander XG MRI Audio System, Resonance Technology inc.), and a “quiet” acquisition approach were used to minimize the interference effect of scanner noise during fMRI [11]. Anatomical images were collected using a T1-weighted 3D-MDEFT sequence [12] (TE/TR = 7/15 ms, 0.94×0.94×1.00 mm^3^ spatial resolution, axial orientation, 256 readout and 192×96 phase-encoding steps, 16 minutes scan time) and a modified T2-weighted Hyperecho sequence [13] (TE/TR = 0.042/10 seconds, echo train length = 16, 256×256 matrix size, 30 coronal slices, 0.86×0.86 mm^2^ in-plane resolution, 5 mm thickness, no gap, 2 min scan time), and reviewed by the neurologist to rule out gross morphological abnormalities of the brain.

### fMRI analysis

Image reconstruction was performed using an iterative phase correction method in IDL that minimizes signal-loss artifacts in EPI [14]. The first four imaging time points were discarded to avoid non-equilibrium effects in the fMRI signal. The statistical parametric mapping package SPM2 (Welcome Department of Cognitive Neurology, London UK) was used for subsequent analyses. A 4^th^ degree B-spline function without weighting and without warping was used for image realignment (head motion was less than 1-mm translations and 1°-rotations for all scans for all fMRI runs). Spatial normalization to the Talairach frame of reference was performed using a 12-parameters affine transformation with medium regularization, 16-nonlinear iterations and voxel size of 3×3×3 mm^3^, and a modified version of the standard SPM2 EPI template, which was modulated by the average EPI signal intensity across subjects to minimize the effect of brain regions exhibiting strong susceptibility-related signal-loss artifacts. Note that this customized EPI template minimizes spurious geometric distortions during spatial normalization of EPI datasets collected at 4-Tesla and TE = 20 ms. An 8-mm full-with-half-maximum (FWHM) Gaussian kernel was used for spatial smoothing.

BOLD-fMRI responses during GD1 and GD2 were estimated using a general linear model [15] with two independent castle designs. As in our previous work [3], six conditions were used for GD1: (1) ratings; (2) flow in and volume < 250 ml; (3) flow in and 250 ml < volume <500 ml; (4) null flow and volume = 500 ml; (5) flow out and 250 ml< volume <500 ml; and (6) flow out and volume <250 ml. Paralleling GD1, the design matrix for GD2 had 8 conditions: (1) ratings; (2) flow in and volume <250 ml; (3) flow in and 250 ml< volume <500 ml; (4) flow in and 500 ml< volume <700 ml; (5) null flow and volume = 700 ml; (6) flow out and 500 ml < volume <700 ml; (7) flow out and 250 ml< volume <500 ml; and (8) flow out and volume < 250 ml. These design matrices were convolved with a canonical hemodynamic response function (HRF) and high-pass filtered with frequency cut-offs of 1/500 and 1/700 Hz for GD1 and GD2, respectively. The BOLD signal strength was estimated without the removal of global effects (global normalization) to minimize false deactivation signals [16], [17].

### Statistical analyses

We evaluated the effect of BMI on BOLD-fMRI signals in the whole brain with a multiple regression analysis in SPM2. Specifically, the estimated BOLD signal maps for all volumetric conditions (0–250 ml, 250–500 ml, 500 ml, 500–700 ml, and 700 ml), subjects, and sessions (GD1 and GD2; first and second repetition) were included in a multiple regression (with constant) random-effects model in SPM2 with two regressors; (1) a zero-mean regressor reflecting the average volume of each condition (125 ml, 375 ml, 500 ml, 600 ml, and 700 ml), and (2) a zero-mean regressor reflecting the subjects' BMI. These multiple regression analyses were conducted using 309 images. Brain activation clusters were corrected for multiple comparisons using the continuous random field calculation implemented in SPM2. A family-wise error (FWE) threshold P*_corr_*<0.05, corrected for multiple comparisons at the voxel-level, was used to display statistical maps reflecting correlations of brain activation and BMI. Clusters with at least 5 voxels and *P_corr_*<0.05, corrected for multiple comparisons, were considered significant in group analyses of brain activation.

### Functional ROI-analyses

Brain activation clusters were further evaluated with region-of-interest (ROI) analyses to identify potential outliers that might influence linear regressions, and to report average values in a volume comparable to the image smoothness (e.g. resolution elements, or “resels” [18]) rather than single-voxel peak values. The volume of the resels was estimated using the random field calculation in SPM2 as a near cubic volume with Cartesian FWHM = 13.1 mm, 12.7 mm, 12.7 mm. Thus, 9-mm isotropic masks containing 27 imaging voxels (0.73 ml) were defined at the centers of relevant activation clusters to extract the average % BOLD signal from individual contrast maps. These masks were created and centered at the precise coordinates listed in Table 1; the coordinates of the ROI masks were kept fixed across subjects and conditions. The average and standard deviation values of BOLD signals within these ROIs were computed for each subject and fMRI run using a custom program written in IDL.

**Table 1:** Location of major activation clusters in the Talairach frame of reference and statistical significance of brain activation for gastric distention (GD: GD1 and GD2, conjunctive analysis), body mass index (BMI), as well as for the cross-correlation, ρ, of fMRI signals in these ROIs with signals in hypothalamus.

ROI	Brain region	BA	*x* [mm]	*y* [mm]	*z* [mm]	GD T-score	BMI T-score	ρ T-score
	**Activated cortical regions**							
1	Inferior parietal cortex	40	−51	−48	51	**9.3**	NS	NS
2	Precentral gyrus	4	−39	−24	66	**9.7**	NS	NS
3	Posterior insula	13	−42	−3	−3	**5.2**	6.3	−6.9
4	Posterior insula	13	36	−3	0	**4.8**	NS	NS
5	Supramarginal gyrus	40	−39	−39	36	**3.8**	6.1	NS
6	Precuneus	31	−15	−57	36	**4.8**	7.3	NS
7	Paracentral Lobule	5	−9	−42	54	**4.5**	6.1	NS
	**BMI sensitive brain regions**							
8	Cerebellum (Declive)		−9	−63	−21	2.9	**8.8**	−3.2
9	Cerebellum (Tonsil)		30	−60	−33	3.5	**8.2**	−3.7
10	Cerebellum (Uvula)		−24	−66	−24	NS	**8.0**	−6.9
11	Thalamus (Ventral Lateral)		−9	−9	3	−2.6	**−5.4**	13.9
12	Anterior Insula	45–47	−36	36	6	−2.7	**−6.1**	5.2
13	Anterior Insula	45–47	48	33	−3	NS	**−6.8**	NS
14	Hypothalamus		−9	−6	−3	NS	**−4.5**	17.6
15	Midbrain		−9	−15	−6	NS	**−6.1**	10.7
16	Midbrain		6	−15	−6	NS	**−5.0**	18.1
17	Amygdala		−21	−12	−12	7.2	**−6.5**	4.2
18	Pons		−3	−18	−27	−2.9	**−5.1**	NS
	**Deactivated brain regions**							
19	Precentral gyrus	6	−39	0	30	**−8.0**	NS	NS
20	Inferior Frontal gyrus	9	33	9	27	**−9.9**	−4.4	3.1
21	Superior Frontal Gyrus	6	15	9	63	**−9.2**	NS	NS
22	Caudate		15	9	6	**−11.2**	NS	4.3
23	Caudate		−6	12	3	**−7.5**	NS	2.8
24	Anterior Cingulate Gyrus	24	0	27	0	**−7.7**	NS	−6.0
25	Parahippocampal gyrus	30	15	−36	−3	**−7.8**	−3.5	NS
26	Lingual gyrus	19	−15	−48	−3	**−6.6**	NS	NS
27	Cerebellum (culmen)		−24	−39	−24	**−11.6**	−7.5	NS

Average values in isotropic cubic ROIs (27 voxels; 0.73 cc) centered at the (*x*, *y*, *z*) coordinates. Sample size = 24 healthy subjects.

#### Functional Connectivity

For each subject and fMRI run, the realigned, normalized, and smoothed time-varying MRI signals within the ROIs previously defined and listed in Table 1 were band-pass filtered (0.01–0.1 Hz frequency bandwidth); then, the Pearson product-moment correlation coefficient was used to calculate the cross-correlation between signals in different ROIs. The Fisher transform was used to normalize the step distributed correlation coefficients, *R_ij_*, of the functional connectivity matrix. Thus, the probability density function of the normalized cross-correlation coefficients, ρ*_ij_*, was approximately Gaussian, allowing us to analyze the statistical significance of the functional connectivity matrix across subjects using standard statistics (*t*-tests). Normalized cross-correlation coefficients with *P*_c_<0.05, corrected for multiple comparisons (Bonferroni correction for 351 comparisons), were considered significant in group analyses of functional connectivity.

## Results

### Behavior

**Figure 2:**
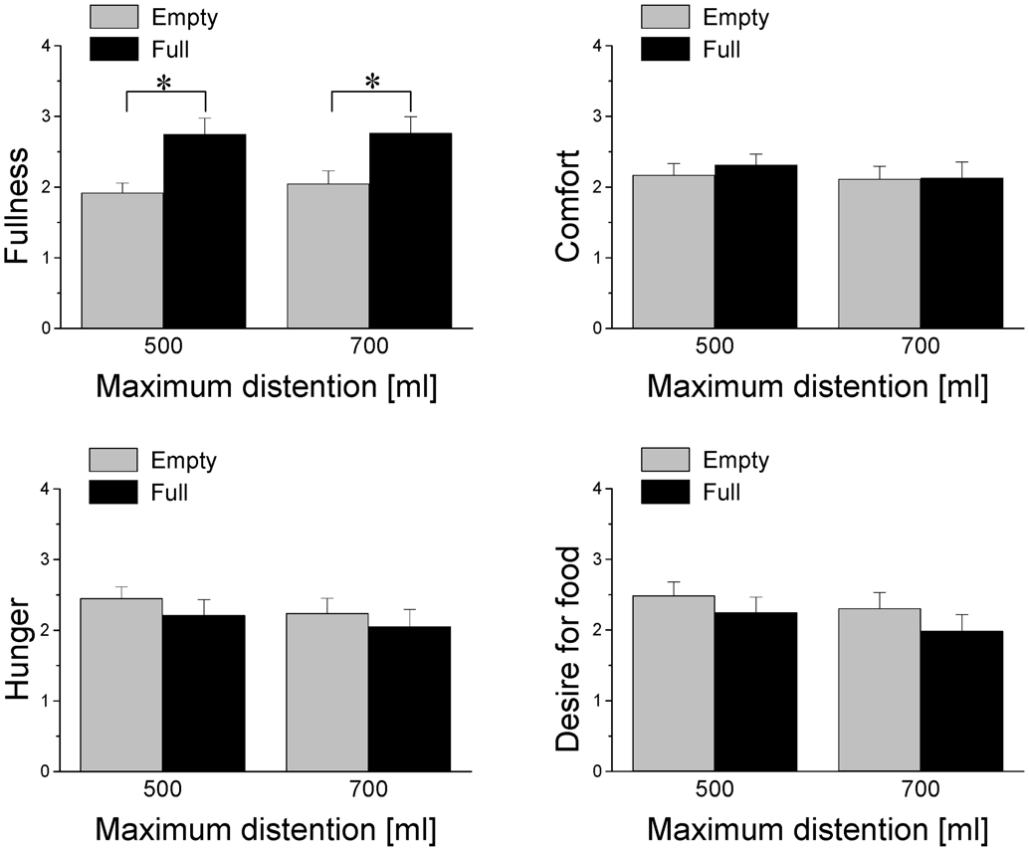
Behavioral responses during gradual GD. Ratings of fullness, discomfort, hunger, and desire for food, collected during the empty and full balloon conditions for GD1 (500 ml) and GD2 (700 ml). (*) P<0.0002.

Ratings of fullness, discomfort, hunger, and desire for food, collected during the empty and full balloon conditions were averaged across fMRI runs, independently for GD1 and GD2 and for each subject; the rating datasets corresponding to 4 subjects were lost due to data acquisition problems. For GD1 and GD2, the rating of fullness was significantly higher for the full condition than for the empty condition (*P*<0.0002; paired t-test; Fig. 2). There were no statistically significant differences in other rating variables between full and empty conditions. There were no significant correlations between BMI and none of the rating variable.

### fMRI: Brain activation

**Figure 3:**
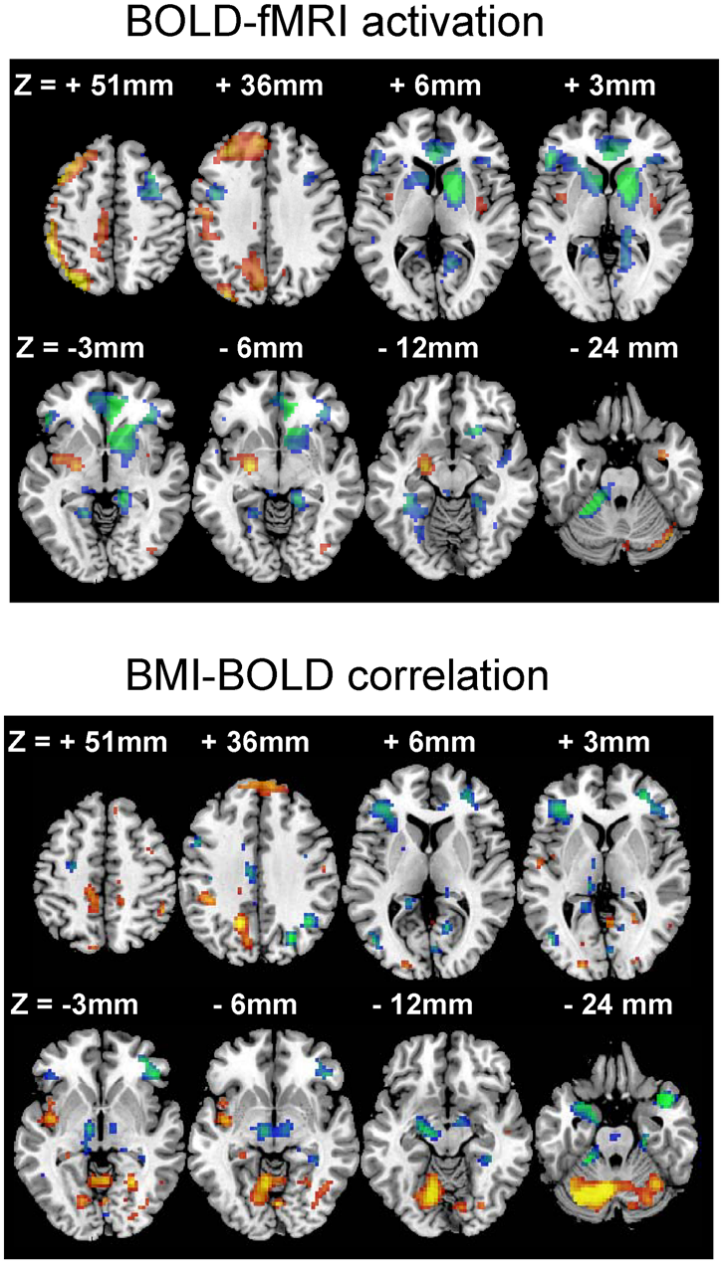
Brain activation during gradual gastric distention (GD). Top panel: Statistical maps showing regions with significant brain activation (red-yellow) and deactivation (blue-green) during gradual GD. Bottom panel: correlations between BMI and BOLD-fMRI responses in the brain during gradual GD. Threshold for statistical significance: *P*_corr_<0.05 corrected for multiple comparisons using the family-wise error (FWE) correction. Sample: 24 healthy controls. Data from all 47 GD1 and 43 GD2 fMRI runs were included in SPM2 multiple regression analyses. Activation/correlation patterns reflecting the effect of volume were not significant in any brain region.

Two subjects did not tolerate the 700 ml balloon distention paradigm (2 GD2 runs per subject); only the GD1 runs were included in the analysis for these subjects. One GD1 dataset and one GD2 dataset were lost due to technical problems. Therefore, a total of six fMRI runs were not completed; thus, 47-GD1 and 43-GD2 fMRI runs were included in the analyses. The GD paradigm (conjunctive analysis of GD1 and GD2) caused bilateral activation in insula, left inferior and superior parietal, and left prefrontal cortices, left precuneus, left amygdala and right cerebellum [*P*_corr_<0.001, corrected for multiple comparisons using the family-wise error (FWE) threshold p = 0.05; Fig. 3 top panel]. This activation pattern is similar to that reported in our prior study for GD1 and a smaller sample that did not include obese subjects [3]. Furthermore, the conjunctive analysis of GD1 and GD2 revealed negative BOLD responses (brain deactivation) to GD bilaterally in caudate, anterior cingulate, parahippocampal, and lingual gyri, as well as inferior and superior frontal gyri and left cerebellum (culmen). Brain activation/deactivation changes resulting from increased balloon volume (from 500 to 700 ml) were not statistically significant in any brain region.

### BMI vs. brain activation

Increased BMI was linearly associated with higher bilateral activation in cerebellum (declive, tonsil and uvula) and left posterior insula and lower bilateral activation in thalamus (dorsal lateral nucleus), anterior insula, midbrain (substantia nigra), amygdala, pons (dorsal raphe), and in a sub-thalamic gray matter region at the location of the posterior hypothalamus [19] (*P*_corr_<0.001; Fig. 3: bottom panel). As shown in Table 1, the positive correlation patterns overlapped GD-activation in left parietal and temporal regions including supramarginal gyrus (BA 40), precuneus (BA 31), and paracentral lobule (BA 5). The negative correlation pattern overlapped GD-activation in amygdala and GD-deactivation in inferior frontal (BA 9) and parahippocampal (BA 24) gyri and left cerebellum (culmen).

### Region-of-interest analyses

**Figure 4:**
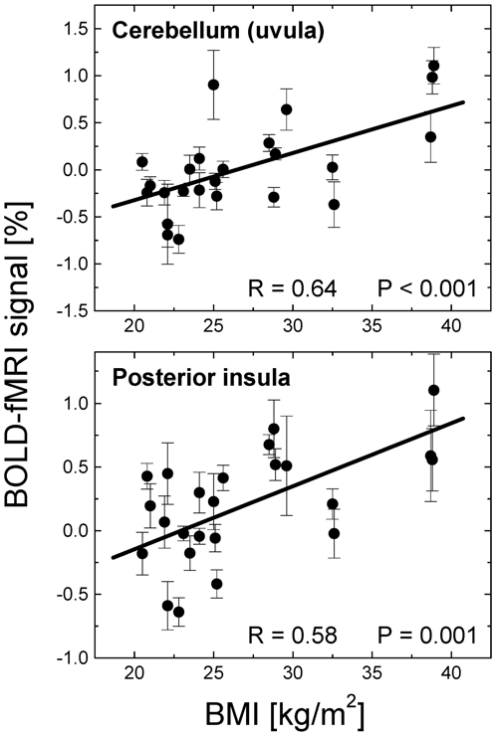
BOLD signals in hyper-activated regions vs. BMI. Scatter plots exemplifying the positive correlations between the body mass index (BMI) and the average BOLD-fMRI response across all volumetric conditions (125, 375, 500, 600, and 700 ml) in cerebellum and posterior insula during gradual GD (N = 24).

**Figure 5:**
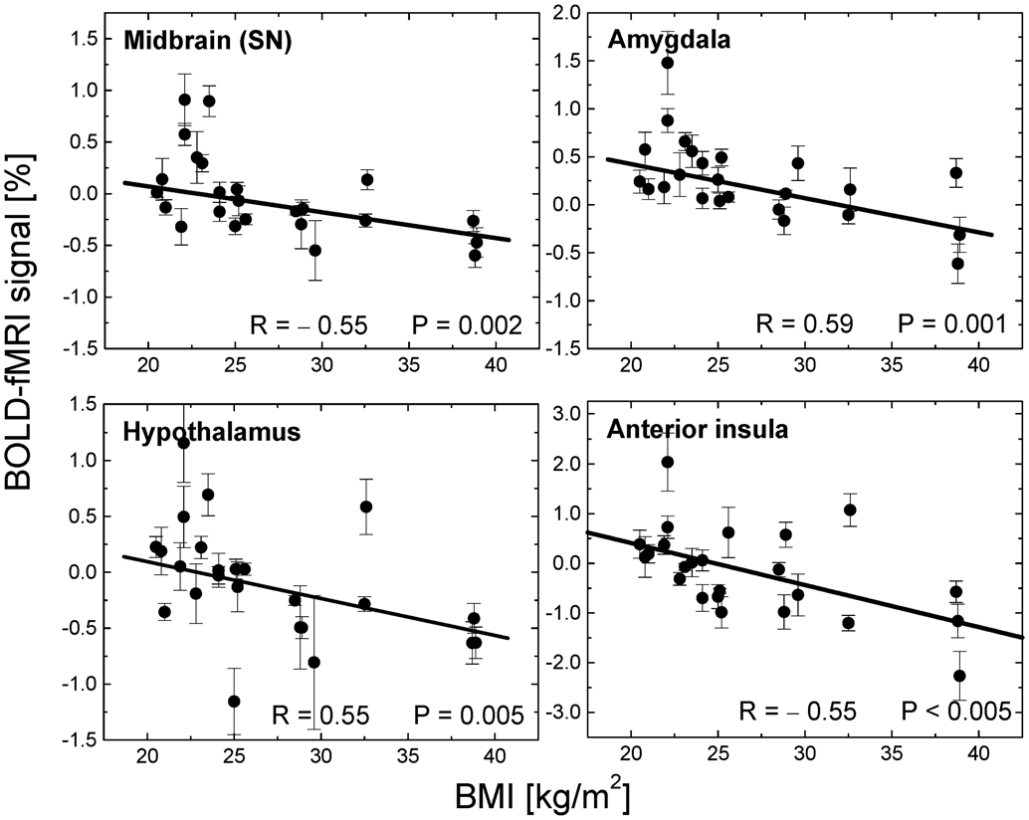
BOLD signals in hypo-activated regions vs. BMI. Scatter plots exemplifying the negative correlations between BMI and the average BOLD-fMRI responses across all volumetric conditions (125, 375, 500, 600, and 700 ml) in dopaminergic brain regions (hypothalamus, midbrain, and amygdala) during gradual GD (N = 24).

**Figure 6:**
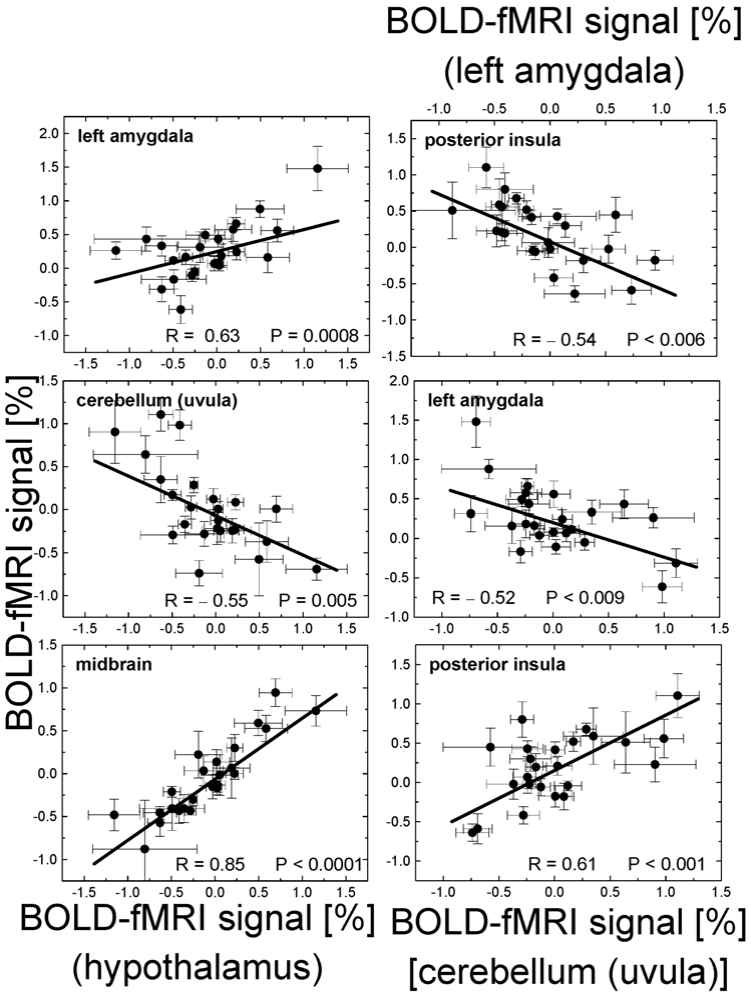
Association of BOLD signals in hyper- and hypo-activated regions. Regression plots exemplifying positive and negative cross-correlations of BOLD-fMRI signals (averaged across all volumetric conditions; 125, 375, 500, 600, and 700 ml) in different ROIs (listed in Table 1). Sample = 24 subjects.

The ROI analyses were consistent with the voxel-wise SPM results in all regions (Table 1). Figures 4 and 5 exemplify the positive and negative correlations of brain activation with BMI across subjects. Indeed, BOLD responses in left posterior insula, supramarginal gyrus, paracentral lobule, and cerebellum (declive, tonsil, and uvula) were significantly higher for obese than for lean subjects (*P*<0.04; Fig. 4). Conversely, BOLD responses in anterior insula, thalamus, hypothalamus, midbrain, amygdala and pons were significantly higher for lean (N = 11) than for obese (N = 5) subjects (*P*<0.05; Fig. 5). Averaged across all volumetric conditions, the BOLD-fMRI responses in the hypothalamus were positively correlated across subjects with those in amygdala, and midbrain and were negatively correlated with those in cerebellum (|R|>0.55; P<0.005; Fig. 6); similarly, responses in cerebellum (uvula) were positively correlated across subjects with those in posterior insula as well as in other regions of the cerebellum (tonsil and declive) (R>0.70; P<0.0002).

### Functional Connectivity

**Figure 7:**
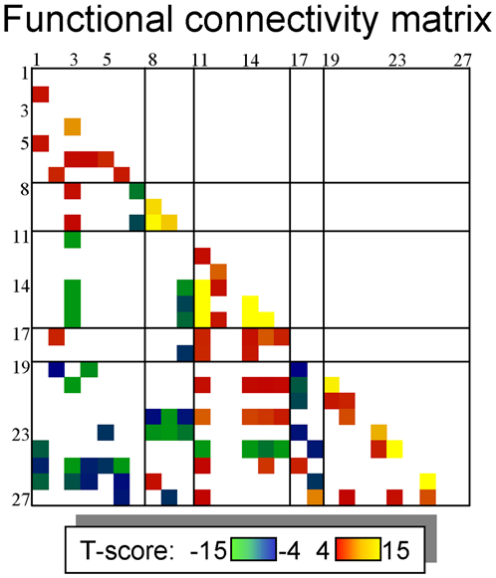
Functional Connectivity during gastric distention. Statistically significant cross-correlations among time-varying fMRI signals in the 27 ROIs listed in Table 1; Sample: 24 subjects, 90 fMRI runs; t-test; statistical threshold *P*_c_<0.05 (Bonferroni correction for 351 comparisons).

Figure 7 shows the statistical significance of normalized cross-correlations of time-varying fMRI signals among the 27 ROIs listed in Table 1 (*t*-test across subjects and fMRI runs). Four major cross-correlation patterns can be highlighted: First, BOLD-fMRI signals in brain areas that exhibited negative BMI-BOLD correlation (thalamus, anterior insula, hypothalamus, midbrain, amygdala and pons) were positively cross-correlated; signals in these brain regions had positive cross-correlations with those in deactivated brain regions (left precentral and lingual gyri, right parahippocampal and well as inferior and superior frontal gyri, and caudate), and negative cross-correlations with those in cerebellum (uvula), left posterior insula, and anterior cingulate gyrus. Second, signals in amygdala and pons had negative cross-correlation with those in most deactivated regions (superior prefrontal cortex, caudate, and anterior cingulate gyrus). Third, signals in left parietal, temporal, and prefrontal regions had negative cross-correlation with those in most deactivated brain regions. Fourth, signals in caudate had negative cross-correlation with those in cerebellum.

## Discussion

GD is thought to play an important role in the regulation of food intake. Here we document that during GD, brain activation responses in cerebellum and left posterior insula had a positive correlation with BMI whereas those in anterior insula, thalamus, amygdala, posterior hypothalamus, midbrain, and pons had a negative correlation with BMI. Inasmuch as BMI reflects in part individual's eating behaviors, the opposite BMI-BOLD correlations suggest that activation in these regions reflects opposing processes involved in the modulation of food intake as a function of GD.

### Negative correlations with BMI: midbrain, hypothalamus, amygdala, thalamus, anterior insula and pons

Here we report that GD-activation in midbrain (where most dopamine neurons are located [20]), pons (where serotonergic nuclei involved in the modulation of GD are located [21]), hypothalamus (a brain region involved in the control of food intake [22]), amygdala (limbic region implicated in emotional reactions to food [23], [24]) and thalamus (brain region implicated in arousal [25], [26]) was negatively correlated with BMI. Furthermore, BOLD-fMRI signals in these regions were highly cross-correlated with one another. The GD paradigm activated midbrain, pons, amygdala, thalamus, and hypothalamus in lean subjects but not in obese subjects. The negative correlation of BMI with BOLD responses in midbrain, is compatible with the role of dopaminergic modulation of food intake whereas that in the pons with the role of serotonin in the regulation of food intake [27], [28]. Indeed appetite suppressants such as phentermine [29] increase both DA as well as serotonin in rats, and there is evidence of both dopaminergic [9] and serotonergic deficits [30], [31] in obese subjects. Thus the decreased GD-activation in midbrain and pons for obese subjects could reflect decreased sensitivity of dopaminergic and serotonergic neurons to vagal stimulation. The hypothalamus, thalamus and amygdala, which were other brain regions showing a negative correlation with BMI, receive dopaminergic [32]–[38] and serotonergic innervations [39]–[41] and thus their decreased activation in obese subjects could also reflect decreased dopaminergic and serotonergic neurotransmission in obese subjects. Moreover the positive correlations between the BOLD signals in pons and midbrain with those in hypothalamus, thalamus and amygdala support the notion that activation in these regions reflects an interconnected pathway, the response of which is modulated in part by DA and serotonin neurotransmission. We had previously reported activation of the left amygdala during gradual GD [3] and here we expand to uncover a negative association between BMI and activation in amygdala, and a correlation between activation signals in amygdala with those in hypothalamus and cerebellum. The latter is likely to reflect the connection of the amygdala with the cerebellum via the hypothalamus [42]. The amygdala, is considered the “sensory gateway to emotions”, and receives sensory information from the forebrain [43] and from the gut [44]. The amygdala plays an important role in feeding behavior [45], [46] and amygdala lesions can result in hyperphagia, and excessive weight gain [23], [24]. The negative association of BMI with activation in hypothalamus is also consistent with the preeminent role of the posterior hypothalamus in regulating eating behaviors and body weight [22]. Indeed, a prior study showed that hypothalamic deactivation after oral glucose administration was markedly attenuated in obese subjects [47] and our findings further suggest abnormal hypothalamic sensitivity to vagal stimulation in obesity.

Activation in pons in an area that included the superior gray matter subnucleus of the dorsal raphe was also negatively associated with BMI; obese subjects deactivated this region whereas lean subjects activated it. Serotonergic neurons in the dorsal raphe are recognized to play an important role in food intake [21]. Dorsal raphe neurons project to the dorsal vagal complex, which mediates vagal stimulation of gastric motor function in rats [48], [49]. Thus, the increased deactivation of the pons with increased BMI during gradual GD, and its positive functional connectivity with midbrain, hypothalamus, anterior and posterior insula, and thalamus suggests functional interactions between dopaminergic and serotonergic signals in the modulation of food intake with GD.

In addition we also show that activation of the anterior insula was negatively correlated with BMI. The anterior insular cortex is implicated in awareness [50]. Thus, the negative correlation between BMI and BOLD-fMRI responses in anterior insula suggests that subjects with higher BMI had lower awareness of the balloon in the stomach than those with lower BMI. Our findings differ from those that showed anterior insula deactivation in lean subject but activation in obese subjects after food intake [51] or during high-calorie food stimulation [52]. This opposite correlation pattern with BMI (negative in our study with GD and positive with exposure to food stimuli) is likely to reflect the differences in stimulation paradigms. The anterior insula is activated by taste perception [53], [54] and food stimulation [55] and the enhanced activation in obese subjects may reflect increased sensitivity to food stimuli whereas the blunted activation to GD may reflect their decreased sensitivity to vagal signaling from GD.

### Positive correlations with BMI: cerebellum and posterior insula

The GD paradigm activated cerebellum and posterior insula, consistently with previous studies on GD that used sudden [5]–[7] or gradual [3] balloon volume changes. Here we document for the first time that activation of cerebellum and posterior insula was proportional to BMI. Moreover, GD activated these regions in obese subjects but not in lean subjects. The cerebellum has an important role for numerous nonsomatic functions other than motor control, and there is growing evidence implicating the cerebellum in the regulation of visceral functions and feeding control [56]. The cerebellum is directly connected to the hypothalamus, a brain region that regulates food intake, energy homeostasis, and body weight [57], and there is evidence of cerebellar modulation of feeding-related hypothalamic neurons [58]. Therefore, it has been suggested that the cerebellar-hypothalamic pathway has an important role in food intake [59]. Moreover, cerebellectomy in rodents resulted in reduced food intake when compared with sham-operated animals, which supports a feeding promoting role of the cerebellum in food control [60]. Previous fMRI studies have shown that the cerebellum activates in response to gustatory or olfactory stimulation [61], [62], during high-calorie vs. low-calorie visual stimulation [63], and 1-minute after oral glucose intake [64]. Positron emission tomography (PET) studies have shown that increased regional cerebral blood flow (rCBF) in cerebellum was associated with hunger and appetite [65], whereas decreased rCBF has been linked with satiation [66], [67]. Prior imaging studies have also documented differences in rCBF in cerebellum after food intake between obese and lean men [66], thus supporting the importance of this brain region in the neuropathology of obesity.

The posterior insula, which was the other brain regions showing a positive correlation with BMI, is activated by taste perception [53], [54], food stimulation [55] and somatic and visceral-sensory processing (reviewed in [68]). The posterior insula is connected with primary and secondary somatosensory cortices and receives inputs from the hypothalamus [69] and the amygdala [70]. Its differential activation as a function of BMI is consistent with a prior study that showed that whereas obese men activated the posterior and middle insula (region overlapping the area where we show positive correlations with BMI) upon exposure to a meal, lean subjects deactivated the middle insula and showed no responses in the posterior insula [71].

### Activation of left cortical regions by GD

Activation responses to GD in ventral parietal cortex and posterior insula as well as somatosensory and motor areas were left lateralized. Previous studies on painful gastric fundus distention have shown either larger activation responses in the left somatosensory cortex than those in the right somatosensory cortex [72], or right lateralized responses in somatosensory and prefrontal cortices [7] and parietal areas (BAs 5, 7, 39, and 40) [5]. The left lateralized response with GD is supportive of a model that proposes lateralization of autonomic functions in the forebrain with left representation of parasympathetic activity (including vagal stimulation) and right representation of sympathetic autonomic activity [73]. However further work is required to properly map the lateralization of autonomic functions in the human brain including those involved with GD.

#### Summary

Here we studied brain activation to GD as a function of BMI. We show BOLD-fMRI responses in dopaminergic (midbrain, hypothalamus, amygdala, thalamus) and serotonergic (pons) brain regions that correlated negatively with BMI, which is consistent with disruption of dopaminergic and serotonergic signaling in obesity. In contrast BOLD responses in posterior insula and cerebellum correlated positively with BMI, which suggests an opposing mechanism that promotes food intake in obese subjects that my underlie their ability to consume at once large food volumes despite increasing GD.
